# A Brief Report of One Hundred Cases of Rötheln

**Published:** 1881-08

**Authors:** Roswell Park

**Affiliations:** 1558 Wabash Ave.; Chicago


### ARTICLE II.

### A BRIEF REPORT OF ONE HUNDRED CASES OF RÖTHELN. By ROSWELL PARK, A.M., M.D., Chicago. (Read before the Illinois State Medical Society, May 17, 1881.)

It is not my purpose to treat in a general way of rötheln, but simply to speak of the peculiar features of an outbreak of the disorder in the Protestant Orphan Asylum of this city, of which I have professional charge. The disease had prevailed to a small extent in scattered localities through the city, and had been recognized as such, about a month before its appearance in the above institution. The first of my cases was diagnosed about the 8th of April, and during the five weeks following, ninety-five children out of one hundred and forty, and two adults, manifested the usual symptoms of this exanthem. These, with a dozen or more cases in private practice, swell the number under my observation to more than a hundred.

Many circumstances rendered it difficult to make out a definite period of incubation, because I could not trace the contagion with sufficient reliability. One feature is worthy of attention first of all, and that is, that only two-thirds of the children exposed were attacked. This is analogous to what we see in the case of the other exanthems, scarlatina for instance; of three children exposed only two may take the disease.

Another point worth mentioning is, that most of these children had already had measles the previous winter or spring; for, during the early months of 1880, eighty-eight children in the same institution were attacked with morbilli.

There were no definite premonitory symptoms. Occasionally the patient would be languid or fretful for a few hours before the eruptive outbreak, and a very few coughed a little, but not in any way to attract particular attention. Along with the appearance of the eruption about twenty-five per cent. presented symptoms like those of the outset of measles. In ten per cent. of the whole number the bronchial irritation, conjunctival suffusion and general appearance were so characteristic that if these same children had not had measles to my certain knowledge, I should have been tempted to diagnose them as such.

The papillary eruption needs no description. I may say, however, that in three or four cases there was a slight tendency for it to become confluent. It appeared first on the neck, throat or behind the ears, traveling downwards, and was usually very distinct on the forearm and wrist. With the appearance of the first papules there was, in almost every case, so much flushing of the cheeks that any manifestation there was hidden from view. The same papules were also plainly visible in the roof of the mouth even before they were distinct on the surface.

Twenty per cent. of the cases had pharyngitis or tonsillitis, but in mild form. Fully one half of them had marked adenopathy in the cervical region or under the tongue.

While the pulse rate was rather high in some of my little patients, the temperature was never any higher than seemed reasonable under the circumstances. Nausea and vomiting were exceedingly rare.

Not one of them suffered from any complication of importance during the progress of the rötheln. But undoubtedly they were left quite susceptible to dangerous sequelæ. Four were prostrated with pneumonia in severe form, though all recovered; and several others suffered from more or less severe bronchitis or croup. This experience has taught me that there is necessity for no small amount of caution for some time after convalescence. In several cases I noticed a very slight furfuraceous desquamation.

The progress of the disorder was, in all cases, toward recovery, and with little or no treatment. I only advised medication when sympathetic fever ran high, when bronchial irritation was marked, or when the bowels were sluggish.

These cases are, I believe, fair samples of the disease which has visited our city, in an epidemic form, this past spring; and while individual cases have hitherto hardly been rare enough to be considered curiosities, yet the disease in its epidemic features has been new to the younger portion of the profession here. For this reason I am perhaps justified in reporting these cases without further apology.

1558 Wabash Ave., May 15, 1881.

